# Rare *Elizabethkingia anophelis* meningitis case in a Danish male

**DOI:** 10.1099/jmmcr.0.005163

**Published:** 2018-08-09

**Authors:** Hans Linde Nielsen, Irene Harder Tarpgaard, David Fuglsang-Damgaard, Philip Kjettinge Thomsen, Sylvain Brisse, Michael Dalager-Pedersen

**Affiliations:** ^1^​ Department of Clinical Microbiology, Aalborg University Hospital, Hobrovej 18-22, 9000 Aalborg, Denmark; ^2^​ Department of Clinical Medicine, Aalborg University, Aalborg, Denmark; ^3^​ Biodiversity and Epidemiology of Bacterial Pathogens, Institut Pasteur, 25 Rue du Dr Roux, 75724 Paris, France; ^4^​ Department of Infectious Diseases, Aalborg University Hospital, Hobrovej 18-22, 9000 Aalborg, Denmark

**Keywords:** *Elizabethkingia*, *Elizabethkingia anopheles*, meningitis, sepsis, mosquito, whole-genome sequencing, antibiotic treatment, moxifloxacin, rifampicin

## Abstract

**Introduction.:**

*Elizabethkingia anophelis* is a Gram-negative, aerobic, non-motile rod belonging to the family *Flavobacteriaceae*. Over the last 5 years, it has emerged as an opportunistic human pathogen involved in neonatal meningitis and sepsis, as well as nosocomial outbreaks. It has been isolated from the midgut of the *Anopheles gambiae* mosquito, but there is no evidence for a role of the mosquito in human infections, and very little is known regarding the routes of transmission to humans. Recent studies, primarily from South-East Asia, suggest that *E. anophelis*, and not *Elizabethkingia meningoseptica*, is the predominant human pathogen of this genus. However, identification to the species level has been difficult due to the limitations of the current MALDI-TOF MS (matrix-associated laser desorption ionization-time of flight MS) systems for correct species identification.

**Case presentation.:**

Here, we present a rare case of *E. anophelis* meningitis in a Danish male, who had a travel exposure to Malaysia 7 weeks before hospitalization. A multidrug-resistant *Elizabethkingia* species was isolated from blood and cerebrospinal fluid, and genomic sequencing was used to characterize the phylogenetic position of the isolate, which was determined as associated with previously described sublineage 11. The patient was successfully treated with intravenous moxifloxacin and rifampicin for 2 weeks with no major sequelae, but we did not find the source of transmission.

**Conclusion.:**

All clinical microbiologists should be aware of the present limitations of the MALDI-TOF MS systems for correct species identification, and therefore we recommend the use of genome sequencing for the correct identification at the species and sublineage level.

## Introduction

*Elizabethkingia* is a genus of Gram-negative, aerobic, non-motile rods first proposed in 2005 by Kim *et al*. [1], belonging to the family *Flavobacteriaceae. Elizabethkingia* species are considered ubiquitous in nature; however, very little is known regarding the routes of transmission of *Elizabethkingia* to humans [2, 3]. The original genus consisted of *Elizabethkingia meningoseptica* and *Elizabethkingia miricola. E. meningoseptica* is presumably the most recognized species causing hospital-acquired infections, including sepsis in immunocompromised patients and neonatal meningitis [4], whereas *E. miricola* is increasingly being isolated from human infections similar to those caused by *E. meningoseptica*, as well as septic arthritis [5].

In 2011, *Elizabethkingia anophelis* was isolated from the midgut of the *Anopheles gambiae* mosquito [6]. In 2013, Frank *et al*. reported the first case of *E. anophelis* infection in humans, which occurred in the Central African Republic and involved an 8-day-old female with neonatal meningitis, which was considered as nosocomially acquired [7]. The authors confirmed the identification of *E. anophelis* by 16S rRNA sequencing. Sequencing of a second strain from another case of neonatal meningitis from 2006, which was originally identified as *E*. *meningoseptica*, subsequently revealed that this strain was in fact *E. anophelis* [7, 8]. Shortly after the first report, several cases of *E. anophelis* infection have been reported from Hong Kong and Taiwan [9, 10], and *E. anophelis* is now recognized as the dominant *Elizabethkingia* species found in blood cultures [11]. Moreover, two extensive outbreaks have occurred in the Midwestern USA involving 65 cases, with a case fatality rate of 31 % (20/65). The majority of patients had bloodstream infections, but some had *E. anophelis* isolated from other sites, such as their respiratory systems or joints (https://www.cdc.gov/elizabethkingia/outbreaks/) [12].

A common feature for *E. anophelis* infection involves a high percentage of healthcare-associated infections, including sepsis/meningitis in neonates, or cases of sepsis in adults with underlying medical conditions including malignancies, diabetes and chronic obstructive pulmonary disease. Here, we present a rare case of *E. anophelis* meningitis in a Danish male, who had a travel exposure to Malaysia 7 weeks before hospitalization.

## Case report

A 76-year-old Danish male presented to the emergency department at Aalborg University Hospital (Aalborg, Denmark) in May 2017 with right-side otalgia throughout the previous week, and onset of fever and confusion within the previous 24 h. Upon admission, the patient was otherwise healthy, and he used no daily medication. Fifty-three days prior to admission, the patient returned from a 16 day holiday on the east coast of Peninsular Malaysia. Before the journey, he was re-vaccinated against diphtheria, tetanus and hepatitis A. He had not used malaria prophylaxis during his vacation.

On examination, the patient had altered mental status with a Glasgow Coma Score of six, neck stiffness and fever (40.0 °C). In accord with national guidelines on the management of suspected bacterial meningitis, the patient had blood cultures performed and was started on high-dose intravenous (iv) benzylpenicillin (1.8 g every 4 h), cefotaxime (3 g every 6 h) and dexamethasone (10 mg every 6 h). A lumbar puncture of the patient was performed, following a computed tomographic scan of the cerebrum, which had proved normal. Laboratory tests showed a C-reactive-protein level of 273 mg l^−1^, procalcitonin of 10.8 µg l^−1^ and white blood cells of 19.9×10^9^ l^−1^. Cerebrospinal fluid (CSF) analysis revealed pleocytosis with white blood cells of 741×10^6^ l^−1^ (636 polynuclear and 105 mononuclear), a slightly decreased glucose ratio (CSF : serum) of 0.38, an elevated protein level of 1.34 g l^−1^ and lactate of 7.3 mmol l^−1^, and the patient was transferred to the Intensive Care Unit (ICU).

Overnight culture of the patient’s CSF yielded Gram-negative, non-motile, oxidase- and catalase-positive rods. Moreover, a simultaneous positive blood culture (BD BACTEC; Becton Dickinson) had similar findings. MALDI-TOF MS (matrix-associated laser desorption ionization-time of flight MS) (MALDI Biotyper 3.1, Bruker Daltonics Microflex LT, MBT 6903 MSP Library) could not distinguish the colonies between either *E. meningoseptica* (score 2.215) or *E. miricola* (score 2.101), whereas *E. anophelis* was not present in the MALDI library. API 20 E v5.0 (bioMérieux) gave an identification of *E. meningoseptica* with a score of 71.6 % identity (numerical profile: 0042004). The isolate was multidrug resistant and positive for metallo-β-lactamase by using the MBL Confirm kit (Rosco Diagnostica). Antimicrobial-susceptibility testing (AST) was performed by using Etests (bioMérieux) according to the European Committee on Antimicrobial Susceptibility Testing guidelines (http://www.eucast.org). We used the pharmacokinetics/pharmacodynamics (non-species related) breakpoints except for trimethoprim/sulfamethoxazole, in which *Stenotrophomonas maltophilia* breakpoints were used, and for gentamicin and colistin we used *Pseudomonas* species breakpoints, as performed by Eriksen *et al*. [5]. The isolate was susceptible to moxifloxacin (MIC 0.125 mg l^−1^) and trimethoprim/sulfamethoxazole (MIC 0.25 mg l^−1^); intermediately susceptible to amoxicillin/clavulanic acid (MIC 6 mg l^−1^); and resistant to ciprofloxacin (MIC 0.75 mg l^−1^) and all other drugs tested including: ampicillin, cefuroxime, ceftazidime, meropenem, gentamicin, colistin and tigecycline. An Etest for vancomycin showed a MIC of 12 mg l^−1^, but we did not make an interpretation of susceptible or resistant. Etests were not available for piperacillin/tazobactam and rifampicin, but the disc diffusion zone (Neo-Sensitabs; Rosco Diagnostica) for piperacillin/tazobactam was 19 mm and for rifampicin was 24 mm, but as for vancomycin we did not make an interpretation of susceptible or resistant.

To determine the identity of the isolate to the species level, we performed sequencing with the Illumina MiSeq instrument producing 2×300 bp paired-end reads by using a Nextera XT library preparation kit (Illumina). Reads were assembled using CLC Genomics Workbench (version 11) (QIAGEN Bioinformatics) into 105 contigs, N50 (497, 160), total sequence length 4 047 726 bp, with a G+C content of 35.6mol %.

**Fig. 1.**
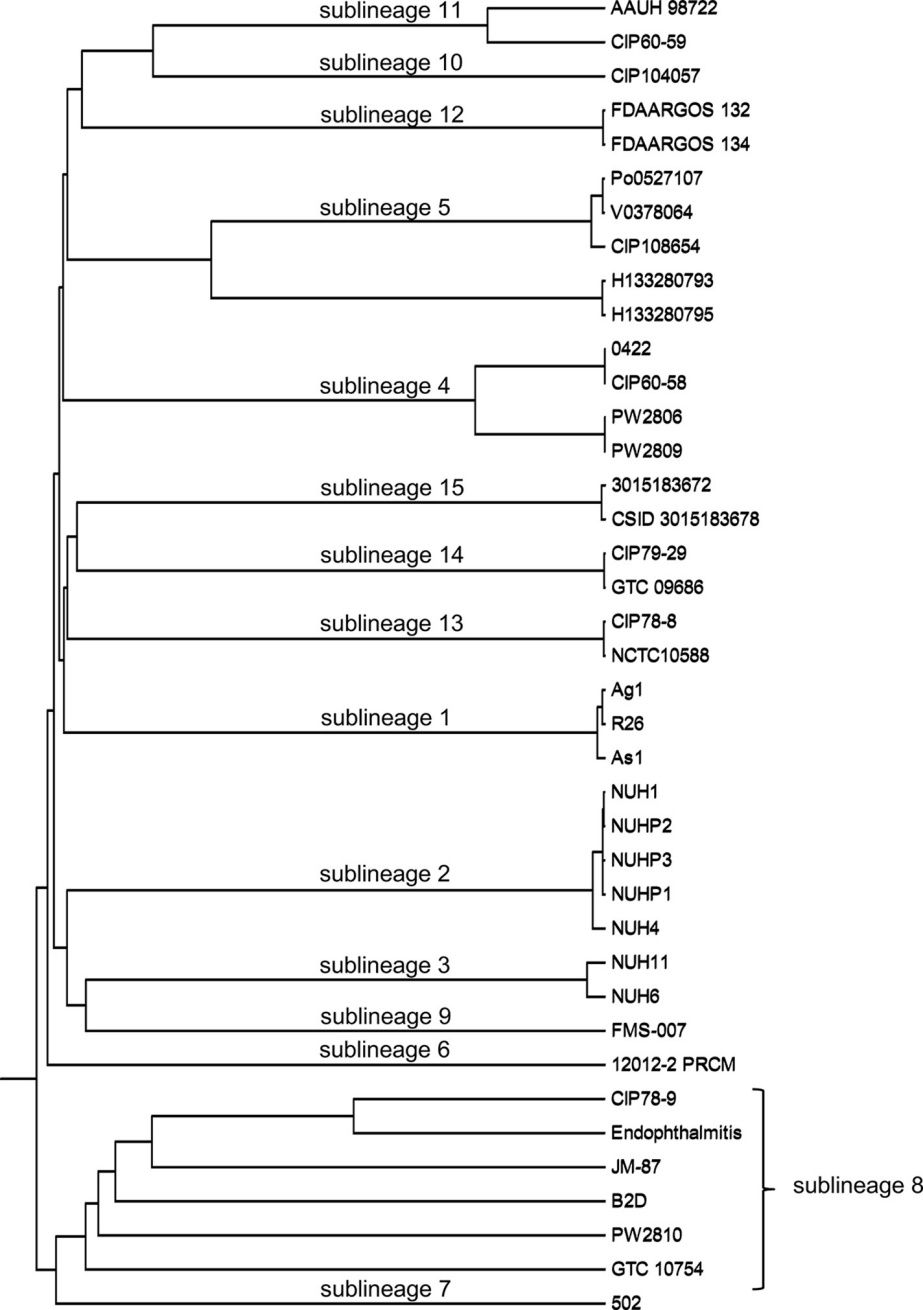
Dendrogram of the *E. anophelis* strains available on the Institut Pasteur server generated by use of BIGSdb software (http://bigsdb.pasteur.fr/elizabethkingia/). The tree was obtained using the UPGMA algorithm based on cgMLST allelic profiles. The scale represents the proportion of loci with distinct alleles among the 1546 gene loci. Our isolate (AAUH 98722) was shown to belong to sublineage 11, which was defined with strain CIP 60-59.

Analysis of the 16S rRNA gene, as well as a k-mer based distance measure, compared to the publicly available strains of *E. meningoseptica* and *E. miricola* showed a clear identification of the isolate as *E. anophelis* (data not shown). Breurec *et al*. [3] and Perrin *et al.* [12] reported a clear division of *E. anophelis* into 15 sublineages, including 1 associated with the large outbreak of *E. anophelis* infections that occurred in Wisconsin (USA) in 2015–2016. To subtype our strain and define its sublineage, we used the core-genome multilocus sequence typing (cgMLST) strategy, using the subset of 1546 genes families that are highly conserved within *E. anopheles* [3]. The cgMLST profile of our isolate was compared to those publicly available in the *Elizabethkingia* cgMLST database on the Institut Pasteur server (http://bigsdb.pasteur.fr/elizabethkingia/). Cluster analysis based on cgMLST profiles, see Fig. 1, showed that the isolate belonged to sublineage 11, which was defined with strain CIP 60–59 (CDC 3375; ATCC 13255; NCTC 10586; CCUG 4321; LMG 12873) as a reference. Strain CIP 60–59 was isolated from the CSF of a premature infant who died [13]; however, the two strains of sublineage 11 have different alleles at 299 loci out of 1513 loci called in both strains. This result clearly shows that AAUH 98722 (our isolate) and CIP 60–59 are genetically distinct. The assemblies were submitted to ResFinder 3.0 (http://cge.cbs.dtu.dk/services/ResFinder/) to analyse them for the presence of antimicrobial-resistance genes [14]. The isolate proved positive for two metallo-β-lactamase genes, namely *bla*_GOB-3_ (located on contig 69; hit length 756; 100 % ID; accession no. AF189291), and *bla*_B-3_ (located on contig 21; hit length 750; 100 % ID; accession no. AF189299), as well as an extended-spectrum β-lactamase-encoding gene, *bla*_CME-1_, (located on contig 41; hit length 784; 100 % ID; accession no. AJ006275). This is consistent with the known conservation of carbapenemase and β-lactamase-encoding genes within *E. anophelis* [3, 12, 15, 16].

After the preliminary identification of *Elizabethkingia* species, the antimicrobial therapy was changed to iv vancomycin combined with iv rifampicin, 600 mg two times a day. However, as MICs values were available the day after, the definite treatment was changed to iv moxifloxacin, 400 mg once per day, combined with iv rifampicin 600 mg two times a day for a total duration of 14 days. The patient improved and after 10 days at the ICU he was transferred to the infectious disease ward for further treatment and rehabilitation, and he was finally discharged after 3 weeks of hospitalization. The only sequela was a partial hearing loss. Due to the severity of infection, the patient was examined for immunodeficiency in an outpatient setting and was found to have a sustained and elevated level of IgM of approximately 14 g l^−1^ (normal range 0.39–2.1 g l^−1^) during the following months. Seven months after the meningitis episode, his bone marrow was further investigated, and he was finally diagnosed with lymphoplasmacytic lymphoma (Waldenström macroglobulinaemia).

## Discussion

Little is known regarding the routes of transmission of *E. anophelis*, and despite investigations by the CDC in the Midwestern USA outbreaks, no potential sources or routes of disease transmission were identified [12]. Our patient had a travel exposure to Malaysia more than 7 weeks before hospitalization, so we cannot rule out the small risk of transmission after exposure to *Anopheles* mosquitoes. However, a long incubation period of 7 weeks seems very unlikely, and phylogenetic analysis showed that the strain was not related to the strains of mosquito midgut origin (Ag1 and R26), and a local acquisition is probably more likely. The patient was cured after 2 weeks of iv moxifloxacin and rifampicin, and suffered a relatively benign sequela in the form of partial hearing loss. However, as the patient was diagnosed with lymphoplasmacytic lymphoma 7 months after his meningitis episode, we cannot rule out that he may have been immunocompromised at the time of diagnosis of *E. anophelis* meningitis.

Previous, correct species identification has been challenged by the limitations of the commercial systems as well as the MALDI-TOF MS systems available on the market, which are unable to identify *E. anophelis* correctly [2], and therefore the latest reports have used 16S rRNA or whole-genome sequencing for the correct identification [7–10]. Recently, Chew *et al*. [11] published a species-specific PCR for the differentiation of *E. anophelis* and *E. meningoseptica*, and it may prove useful in high-epidemic areas like South-East Asia and East Asia.

Our *E. anophelis* isolate was multidrug resistant with production of metallo β-lactamases (*bla*_GOB-3_ and *bla*_B-3_) conferring resistance to third-generation cephalosporins and carbapenems. Fortunately, further resistance testing of the isolate revealed a low MIC to moxifloxacin by Etest and apparent susceptibility to rifampicin by disc diffusion, and the patient was treated successfully with this combination of antibiotics. Levofloxacin, another late-generation fluoroquinolone is, in contrast to moxifloxacin, not available as a treatment option in Denmark. Results from previous studies have shown some discrepancies in sensitivity to later-generation fluoroquinolones. In a study from Singapore, Chew *et al*. found that 78.5 % of 79 isolates from blood were susceptible to levofloxacin [11], whereas a study from South Korea by Han *et al*. determined that 29 and 41 % of isolates were susceptible to levofloxaxcin and moxifloxacin, respectively [17]. Rifampicin was added to the treatment regimen for our patient, and the South Korean data showed 96 % susceptibility to rifampicin among their isolates of *E. anophelis* [17]. However, the Asian studies used other AST protocols and Clinical and Laboratory Standards Institute interpretations [11, 17], and therefore our AST data is not directly comparable.

Vancomycin has been used successfully for treatment of *E. meningoseptica* bacteraemia [18], and initially, the patient was treated with iv vancomycin, in combination with rifampicin. However, the vancomycin level obtained in CSF may be suboptimal, and is highly dependent on inflamed meninges, whereas both moxifloxacin and rifampicin have better CSF penetration that is less dependent on meningeal inflammation [19]. Trimethoprim/sulfamethoxazole could also have been a treatment option for our patient, but recent studies have found variable susceptibility to trimethoprim/sulfamethoxazole [2, 10, 17]. Moreover, trimethoprim/sulfamethoxazole seems to have a higher incidence of adverse reactions compared to moxifloxacin, and that contributed to the selection of moxifloxacin and rifampicin as the targeted treatment.

In conclusion, we present a rare case of *E. anophelis* meningitis in a Danish male. The patient had a travel exposure to Malaysia 7 weeks before hospitalization; however, by phylogenetic analysis and the fact that the isolate belonged to sublineage 11 favours an unknown transmission. The patient was cured by treatment with iv moxifloxacin and rifampicin for 2 weeks. All clinical microbiologist should be aware of the present limitations of the MALDI-TOF MS systems for correct species identification, and therefore we recommend genome sequencing for the correct identification at species and sublineage level.
